# Adiponectin complexes composition in Japanese-Brazilians regarding their glucose tolerance status

**DOI:** 10.1186/1758-5996-5-20

**Published:** 2013-04-09

**Authors:** Felipe Crispim, Marcio F Vendramini, Regina S Moisés

**Affiliations:** 1 Disciplina de Endocrinologia , Escola Paulista de Medicina, Universidade Federal de São Paulo, Rua Pedro de Toledo, 781, São Paulo, SP, 04039-032, Brazil

**Keywords:** Adiponectin, HMW adiponectin, Japanese-Brazilians

## Abstract

**Background:**

Adiponectin circulates in different multimer complexes comprised of low molecular weight trimeric form (LMW), hexamer of middle molecular weight (MMW) and high molecular weight multimers (HMW). In Japanese-Brazilians, a population with high prevalence of glucose metabolism disturbances, we examined the associations of total adiponectin and its multimers with diabetes mellitus.

**Methods:**

Two study groups were examined: 26 patients with diabetes mellitus (DM,14 women and 12 men, aged 55.3 ± 8.6 years) and 27 age-matched control subjects with normal glucose tolerance (NGT,12 women and 15 men, aged 54.0 ± 9.2 years).

**Results:**

We found no significant differences in total [NGT: 6.90 ug/ml (4.38-13.43); DM: 5.38 ug/ml (3.76-8.56), p = 0.35], MMW [NGT:2.34 ug/ml (1.38-3.25); DM: 1.80 ug/ml (1.18-2.84), p = 0.48] or LMW adiponectin [NGT: 2.07 ug/ml (1.45-3.48), DM: 2.93 ug/ml (1.78-3.99), p = 0.32] between groups. In contrast, HMW adiponectin levels were significantly lower in patients with DM [TGN: 2.39 ug/ml (1.20-4.75); DM: 1.04 ug/ml (0.42-1.60), p = 0.001]. A logistic regression analysis was done to identify independent associations with diabetes mellitus. The results showed that HOMA-IR and HMW adiponectin in women were independently associated with diabetes mellitus.

**Conclusion:**

The current investigation demonstrates that in Japanese-Brazilians HMW adiponectin is selectively reduced in individuals with type 2 diabetes, while no differences were found in MMW and LMW adiponectin isoforms.

## Background

Adiponectin, a peptide hormone predominantly expressed and secreted by adipocytes, has been recognized as an important regulator of insulin sensitivity [1-3]. It is present in the circulation of healthy individuals at high concentration, account for approximately 0.01% of total plasma protein. Hypoadiponectinemia is seen in states of insulin resistance such as type 2 diabetes, obesity and metabolic syndrome [4-6]. Adiponectin circulates in different multimer complexes comprised of low molecular weight trimeric form (LMW), hexamer of middle molecular weight (MMW) and high molecular weight multimers (HMW) [7]. Possibly these complexes have different functions and there are evidences indicating that HMW adiponectin might be the most active metabolically form. Indeed, previous studies showed that HMW adiponectin or the ratio HMW to total adiponectin correlates better with measures of insulin sensitivity [8,9] or glucose tolerance [10]. However, other studies failed to demonstrate the superiority of HMW adiponectin over total adiponectin in assessing insulin sensitivity. For example, in a Mexican population total adiponectin, HMW adiponectin and the ratio HMW/total adiponectin had similar utility for the identification of insulin resistance and metabolic disturbances [11]. Similarly, in a longitudinal study with Japanese-Americans lower both total and HMW adiponectin were independent risk factors for the development of metabolic syndrome [12].

Japanese-Brazilian is a population with high prevalence of glucose metabolism disturbances, being the prevalence of diabetes one of the highest worldwide [13]. In a previous study we showed that low plasma level of total adiponectin was an independent predictor for development of glucose intolerance in this population [14]. In this study it was examined the associations of total adiponectin and its multimers with diabetes mellitus.

## Subjects and methods

### Subjects

The study population was selected from the Japanese Brazilian Diabetes Study Group. Details on the selection and recruitment of this population were previously described [13]. For this study, we randomly selected a small subset of non-related subjects with suitable baseline serum specimens and normal glucose tolerance or diabetes mellitus matched for age and sex. Individuals with impaired fasting glucose, impaired glucose tolerance or severe kidney disease (eGFR < 30 mL/min/1.73 m^2^) were excluded. A subset of 53 individuals was selected: 26 patients with diabetes mellitus (DM,14 women and 12 men, aged 55.3 ± 8.6 years) and 27 control subjects with normal glucose tolerance (NGT,12 women and 15 men, aged 54.0 ± 9.2 years). Individuals went through an interview with questionnaires regarding social aspects and medical history. Clinical examination was performed and included anthropometric (weight, height, waist circumference and hip circumference) and blood pressure measurements. Fasting blood samples for glucose, insulin, lipids and adiponectin were obtained. Morning medications were deferred until all procedures were done. A 75 g anhydrous glucose was administered to all subjects with fasting capillary glucose < 200 mg/dL screened by glucose-oxidase strips. After 2 h another blood sample was obtained for glucose measurements.

This study was approved by the Ethics Committee of the Federal University of São Paulo, and all participants gave their informed consent.

### Laboratory methods

Plasma glucose was determined by the glucose-oxidase method. Cholesterol contents of lipoproteins fractions and triglycerides were measured enzymatically. Insulin was determined by a monoclonal antibody-based immunofluorimetric assay (PerkinElmer, Wallac Oy, Turku, Finland) that does not react with proinsulin. Insulin resistance was estimated by homeostasis model assessment (HOMA-IR) [15]. Estimated glomerular filtration rate (eGFR) was determined by the Modification of Diet in Renal Disease (MDRD) equation. Glucose tolerance status was based on WHO criteria [16]. Serum total adiponectin concentrations were measured by radio-immunoassay according to manufacter’s instructions (Linco Research, St. Charles, MI, USA) in specimens that had been stored at −20°C. This assay utilizes ^125^I-labeled murine adiponectin and a multi-species adiponectin antiserum. Samples were diluted 1:500 in assay buffer prior to measurements. The assay has a sensitivity of 1 ng/mL with an intra- and inter-assay coefficients of variation of 1,8-6,2% and 6,9-9,3%, respectively.

### SDS-PAGE and immunoblotting for determination of adiponetin multimers

The relative amounts of adiponectin multimers were determined by Western blot analysis. Serum was diluted 10-fold with deionized water and 5 uL of this diluted serum plus 5 uL of protein loading buffer was separated under non-reducing and non-heat-denaturating conditions on 4-12% polyacrylamide gel electrophoresis. SDS-PAGE was performed according to standard Laemmli procedure. Following electrophoresis, the proteins were transferred to nitrocellulose membrane (Hybond ECL, Amersham Biosciences, Piscataway, NJ, USA) and incubated with blocking solution (5% nonfat milk in Tris-buffered saline with 0.05% Tween 20) for 1 h at room temperature. The blocking solution was removed and replaced with the same solution containing 1:10,000 dilution of a mouse antihuman adiponectin monoclonal antibody (BD Bioscience, San Jose, CA, USA). The incubation was continued overnight at 4°C. After being washed the nitrocellulose was incubated with horseradish peroxidase-conjugated goat anti-mouse immunoglobulins (Santa Cruz Biotechnology, Inc, Santa Cruz, CA, USA) with 1:5,000 dilution for 1 h at room temperature and washed thoroughly. Bands were detected by an enzymatic chemiluminescent Western blot detection system (SuperSignal West Pico Chemiluminescent Substrate, Thermo Fisher Scientific, Rockford, IL, USA) and quantified by densitometry analysis using the Image J software. Relative proportions of adiponectin multimers were calculated by dividing band density by total density in each lane. The absolute adiponetin multimer values were extrapolated by multiplying the relative proportions by the total adiponectin concentrations detected by RIA, assuming that all multimers are immunoreactive on the immunoblot.

### Statistical analysis

Data with normal distribution, tested by Kolmogorov-Smirnov test, are expressed as mean ± SD while variables nonnormally distributed are reported as median (interquartile range). Differences in continuous variables between groups were evaluated by unpaired Student’s test or Mann–Whitney test. The relative proportions of adiponectin multimers between groups were compared by using the Holling T2 test. Univariate associations between variables were investigated by Spearman correlation analysis. Logistic regression model was used to identify independent associations with diabetes mellitus. Model fit was assessed using the Akaike Information Criterion. A P-value of < 0.05 was considered statistically significant.

## Results

**Figure 1:**
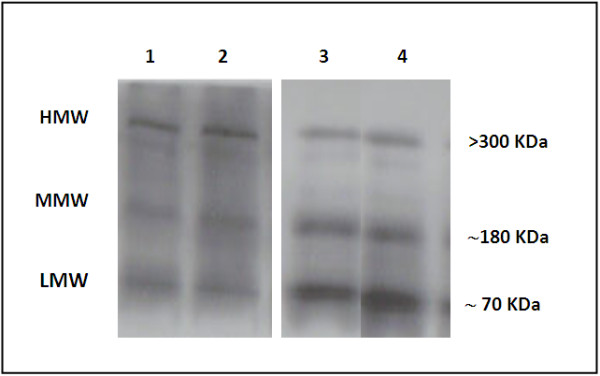
**Representative immunoblot from serum of age and sex matched subjects with NGT (lanes 1 and 2) and DM (lanes 3 and 4).** Band at > 300 kDa represents high molecular weight (HMW) adiponectin, at ~180 kDa represents middle molecular weight (MMW) adiponectin and at ~70 kDa represents low molecular weight (LMW) adiponectin.

**Figure 2:**
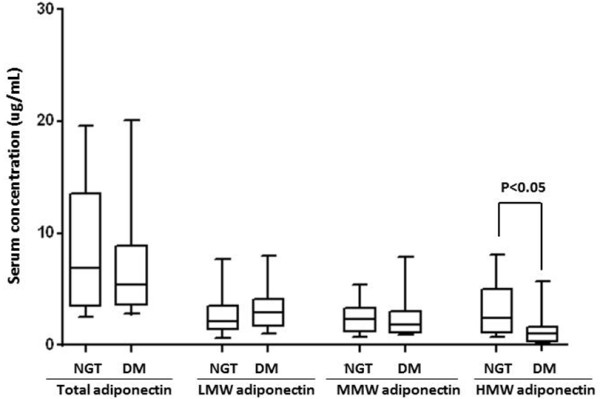
**Serum concentrations of total, low molecular weight (LMW), middle molecular weight (MMW) and high molecular weight (HMW) adiponectin in individuals with normal glucose tolerance (NGT) or diabetes mellitus (DM).** The box displays the median and interquartile range (25th-75th percentile) and the whiskers display the 10th and 90th values.

**Figure 3:**
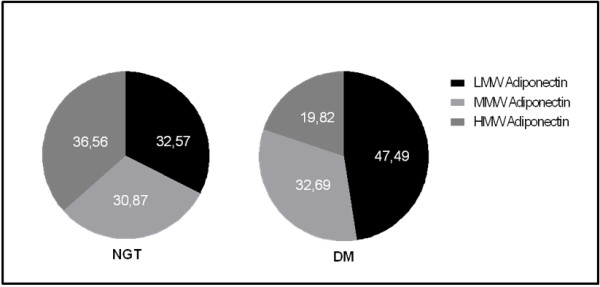
Percentual distribution of low molecular weight (LMW), middle molecular weight (MMW) and high molecular weight (HMW) adiponectin in individuals with normal glucose tolerance (NGT) or diabetes mellitus (DM).

The main characteristics of the study population are shown in Table 1. Individuals with DM had significantly higher BMI, waist circumference, triglycerides and HOMA-IR than subjects with NGT. Figure 1 shows a representative immunoblot from serum of age and sex matched individuals with DM and NGT. Bands were found at > 300 kDa representing HMW adiponectin, at ~180 kDa representing MMW adiponectin and at ~70 kDa representing LMW adiponectin. As displayed in Figure 2, no significant differences in total [NGT: 6.90 ug/ml (4.38-13.43); DM: 5.38 ug/ml (3.76-8.56), p = 0.35], MMW [NGT:2.34 ug/ml (1.38-3.25); DM: 1.80 ug/ml (1.18-2.84), p = 0.48] or LMW adiponectin [NGT: 2.07 ug/ml (1.45-3.48), DM: 2.93ug/ml (1.78-3.99), p = 0.32] were found between groups. In contrast, HMW adiponectin levels were significantly lower in patients with DM [NGT: 2.39 ug/ml (1.20-4.75); DM: 1.04 ug/ml (0.42-1.60), p = 0.001]. The distribution of adiponectin isoforms in subjects with NGT and DM are shown in Figure 3. Lower proportions of HMW adiponectin and higher proportions of LMW adiponectin were observed in subjects with DM compared to NGT. No differences were found in proportions of MMW adiponectin.

**Table 1:** Characteristics of the subjects with normal glucose tolerance (NGT) or diabetes mellitus (DM)

	**NGT (n = 27)**	**DM (n = 26)**	**P value**
Age (years)	54.0 ± 9.2	55.3 ± 8.6	0.58^a^
BMI(kg/m^2^)	23.4 ± 2.4	25.1 ± 2.4	0.015^a^
Waist circumference (cm)	79.4 ± 6.5	85.1 ± 7.6	0.005^a^
Waist-hip ratio	0.83 ± 0.05	0.88 ± 0.07	0.005^a^
Total cholesterol (mg/dL)	200.9 ± 39.3	209.7 ± 33.4	0.384^a^
HDL-Cholesterol (mg/dL)	48.8 ± 14.1	48.1 ± 8.4	0.809^a^
LDL-cholesterol (mg/dL)	121.7 ± 39.1	126.4 ± 33.3	0.638^a^
Triglycerides (mg/dL)	132.0 (97.2-171.5)	227.5 (160.0-380.0)	0.001^b^
Fasting plasma glucose (mg/dL)	104.0 (100.2-107.7)	134.5 (127.0-170.0)	<0.001^b^
Fasting plasma insulin (uU/mL)	5.8 ± 2.7	9.4 ± 3.3	<0.001^a^
HOMA-IR	1.39 (0.96-1.75)	3.01 (2.63-3.83)	<0.001^b^
eGFR (mL/min/1.73 m^2^)	98.1 ± 37.3	97.8 ± 32.8	0.97^a^
HbA1C (%)	-	6.2 (5.7-7.1)	-
Treatment for diabetes (n)			
SU	-	2	-
MTF	-	1	-
SU + MTF	-	1	-
SU + INS	-	1	-
Treatment for hypertension (n)			
B- blocker	1	-	-
Diuretic	1	1	-
ACEi	1	1	-
B-blocker + diuretic	1	1	-
Treatment for dyslipidemia (n)			
fibrate	-	1	-

Data are presented as mean ± SD, median (interquartile range) or n.

SU: sulfonylrea; MTF: metformin; INS: insulin; ACEi: angiotensin-converting enzyme inhibitor;

^a^ t-test.

^b^ Mann–Whitney test.

Spearman rank correlation showed significant inverse correlations between BMI (rs = − 0.29, p = 0.034), HOMA-IR (rs = −0.36, p = 0.007) and triglycerides (rs = −0.22, p = 0.04) with HMW adiponectin, MMW and LMW adiponectin were significantly associated with waist circumference (rs = −0.29, p = 0.03; rs = −0.28, p = 0.03 respectively). A trend for inverse correlation between BMI and total adiponectin (rs = − 0.27, p = 0.051) was observed. No significant correlations were found for total adiponectin with HOMA-IR or triglycerides.

A logistic regression analysis was done to identify independent associations with diabetes mellitus. The covariates were waist/hip ratio, triglycerides levels, HOMA-IR, total and HMW adiponectin, including interaction with sex for total and HMW adiponectin. The results showed that HOMA-IR and HMW adiponectin in women were independently associated with diabetes mellitus (Table 2).

**Table 2:** Odds ratio (OR) and 95% confidence interval (CI) for diabetes mellitus according to biological variables

	**OR**	**95% CI**
HMW adiponectin in women (for each 1%)	0.75	0.58-0.95
HMW adiponectin in men (for each 1%)	1.08	0.95-1.23
HOMA-IR (for each 0.1 unit)	1.53	1.12-2.09

## Discussion

In the present study, we measured total plasma adiponectin and its all three oligomeric isoforms in Japanese-Brazilians to investigate their relationship with diabetes mellitus. There are limited data concerning the role of these oligomers; most studies are mainly based on total adiponectin and HMW isoform.

Different methods have been employed for determination of adiponectin multimers such as velocity sedimentation coupled to quantitative Western-blot analysis, gel filtration chromatography and gel electrophoresis [5,7,17,18]. Pajvani et al. developed a method of velocity sedimentation analysis coupled to Western blot to separate and measure the adiponectin complexes [17]. This is a laborious technique and does not discriminate the lower molecular weight complexes. More recently, Schraw et al. validated a high-resolution gel filtration approach followed by fluorescent Western blotting that separated the three major adiponectin complexes in a reproducibly and accurate manner [19]. Waki et al. using a SDS-PAGE under non-reducing and non-heat-denaturing conditions clearly separated the adiponectin multimers in to three species and reported that this analysis was superior to the gel filtration in terms of resolving power [7]. In this study, it was employed SDS-PAGE under non-reducing and non-heat-denaturing conditions to detect the isoforms. In our hands, this procedure permitted a clear separation of the three bands.

In previous studies we showed that in Japanese-Brazilians, low plasma level of total adiponectin is an independent predictor of glucose intolerance and subjects with type 2 diabetes compared with normal glucose tolerant subjects have reduced levels of total adiponectin [14,20]. Here, in a small subset of this population, it was found that HMW adiponectin is selectively reduced in individuals with type 2 diabetes. As a consequence of a drop in HMW, an increase in the percentage of LMW was observed in individuals with DM compared to NGT. A correlation between insulin sensitivity, evaluated by HOMA-IR, and HMW adiponectin was observed. These findings support the role of HMW adiponectin as the major isoform mediating its insulin-sensitizing effects. Furthermore, in women, an increase of HWW adiponectin in percent has a significant decreased risk of diabetes mellitus. This is in agreement with previous studies showing that decreased HMW adiponectin is a better marker than total adiponectin for assessing the risk of type 2 diabetes [8,21,22]. However, other data do not support the superiority of HMW adiponectin in assessing parameters of insulin resistance [23] or risk of type 2 diabetes [24].

MMW and LMW adiponectin isoforms have not been extensively studied, as a result little is known regarding their distribution. In the present study no differences were found in MMW or LMW adiponectin between groups. Schober et al., using ELISA to measure the three isoforms, found that MMW and LMW adiponectin were reduced in the serum of type 2 diabetic patients when compared to normal weight controls [25]. However, Basu et al. using velocity sedimentation/gel filtration chromatography and Lara Castro et al. using immunoblot to separate HMW form from the remaining adiponectin isoforms found that the concentration of LMW fractions (hexameric and trimeric forms) did not differ in the diabetic and nondiabetic subjects [8,26]. These discrepancies may be explained by different methods for determination of adiponectin multimers or by ethnic differences.

## Conclusions

The current investigation demonstrates that in Japanese-Brazilians HMW adiponectin is selectively reduced in individuals with type 2 diabetes, while no differences were found in MMW and LMW adiponectin isoforms.

## Abbreviations

LMW: Low molecular weight; MMW: Middle molecular weight; HMW: High molecular weight; DM: Diabetes Mellitus; NGT: Normal glucose tolerance; HOMA-IR: Homeostatic model assessment-insulin resistance; BMI: Body mass index; HDL: High density lipoprotein; LDL: Low density lipoprotein; ELISA: Enzyme-linked immunosorbent assay.

## Competing interests

The authors declare that they have no competing interests.

## Authors’ contributions

FC: performed the experiments, analyzed the data and wrote the manuscript; MFV: participated in the design of the study and helped to draft the manuscript; RSM: conceived and designed the experiments, analyzed the data and wrote the manuscript. All authors read and approved the final manuscript.
